# Effects of Serine Acetyltransferase 2 (*sat2*) Haplotypes and Multiple Environmental Factors on Grain Protein Content and Yield-Related Traits in Bread Winter Wheat

**DOI:** 10.3390/plants15213340

**Published:** 2026-10-28

**Authors:** Malak Alkubesi, Alexander Pryanishnikov, Salis Karakotov, Ilya Smith, Ksenia Khurtova, Anastasiya Chernook, Timofei Voinov, Evgenia Obolenskaya, Mikhail Divashuk

**Affiliations:** 1 All-Russian Research Institute of Agricultural Biotechnology (VNIISB), Moscow 127550, Russia; hurtova@list.ru (K.K.); irbis-sibri@yandex.ru (A.C.); divashuk@gmail.com (M.D.); 2 Department of Genetics, Breeding and Seed Science, Russian State Agrarian University—Moscow Timiryazev Agricultural Academy (RSAU-MTAA), Moscow 127434, Russia; scitimeg@gmail.com (T.V.); jenia.obolensckaya@yandex.ru (E.O.); 3 Shchelkovo Agrokhim JSC, Shchyolkovo 141108, Russia; a_pryan@mail.ru (A.P.); info@betaren.ru (S.K.); 4 NPO Betagran Seeds LLC, Dobry Settlement, Oryol 302522, Russia; orelopx@betagran.ru

**Keywords:** *Triticum aestivum*, *sat2*, grain protein content, KASP markers, haplotype, crop management

## Abstract

Serine acetyltransferase 2 (*sat2*), rate-limiting for cysteine biosynthesis, is a candidate gene for grain protein content in durum wheat. We tested whether its intragenic variants are associated with protein content in bread winter wheat and how the effect depends on year, crop management, cultivar origin and other loci. Sixty-five cultivars were genotyped by Kompetitive Allele-Specific PCR for TDsat2.e9.1 (G>A, *Gly325Ser*) and TDsat2.i1.1 (G>T, intron 1). The variants were completely linked: only Hap1 (G; T; 32 cultivars) and Hap2 (A; G; 33 cultivars; the Chinese Spring reference haplotype) occurred. The 5913 observations (2021–2025; 16 year × management environments at one site) were analysed with a linear mixed model, the cultivar being the unit of replication. Hap1 exceeded Hap2 in protein content by 0.58% (95% CI 0.22–0.93; *p* = 0.0017; q = 0.014); the effect persisted with grain yield as a covariate and after adjustment for *Glu-A1*, *Glu-D1* and *1BL.1RS*, and haplotype frequencies were unrelated to cultivar origin and *Rht-B1*. Independently measured traits, including grain yield, did not differ. The effect kept its sign in all years but varied in size (*p* = 0.0013); no interaction with crop management was detected. The association requires confirmation in other collections.

## 1. Introduction

Bread wheat (*Triticum aestivum* L.) is one of the principal food crops of the world and the main source of plant protein for the populations of Europe and Asia [1]. In Russia it is the leading grain crop owing to its yield, adaptability and versatility of use [2].

Gluten is an insoluble complex of storage proteins, mostly glutenins (high- and low-molecular-weight subunits, HMW-GS and LMW-GS) and gliadins; HMW-GS forms its structural framework [3,4]. Gliadin genes lie in the *Gli-1* and *Gli-2* clusters of homoeologous groups 1 and 6 and glutenin genes in the *Glu-1* and *Glu-3* loci of group 1; allele frequencies at these loci differ between regional gene pools and between bread and durum wheat [5,6], and the two species also differ in the gliadin-to-glutenin ratio [7,8].

Regulatory factors act on grain quality alongside the structural genes. The NAC transcription factors Gpc accelerate senescence and nitrogen remobilisation to the grain [9], dwarfing Rht genes alter nitrogen partitioning between grain and biomass [10], the 1BL.1RS translocation improves disease resistance but can impair bread-making quality [11], and photoperiod sensitivity genes shift the timing of grain filling [12]. Weather in a given year affects grain quality on a scale comparable with that of the genotype [13,14].

Sulphur supply acts on grain quality through a separate route. Cysteine, which occurs mainly in glutenins, forms the intermolecular disulfide bridges that determine the elasticity of the gluten network [15,16], so genes of cysteine biosynthesis are candidates both for protein content and for gluten quality. The *sat2* gene encodes serine acetyltransferase, which catalyses the formation of O-acetylserine, the rate-limiting step of cysteine biosynthesis. In durum wheat, a genome-wide association study on 190 accessions identified a locus on chromosome 4B for which *sat2* was proposed as the candidate gene; the gene was sequenced and two polymorphisms were found. The missense variant *Gly325Ser* in exon 9 was associated with an increase in protein content of 1.33% in recombinant inbred lines (*p* = 0.003) and the intron 1 variant with an increase of 0.92% in an association panel of 230 accessions; KASP markers were developed for both [17]. Expression of the bread wheat homolog peaks in the aleurone and sub-aleurone layers 20–30 days after flowering, during storage protein accumulation [17].

Serine acetyltransferase does not act in isolation: its product, O-acetylserine, is converted to cysteine by O-acetylserine (thiol) lyase (OASTL), and the two enzymes form the cysteine synthase complex, whose assembly and dissociation regulate the flux of sulphur into cysteine [18,19,20]. The acetyltransferase step is rate-limiting and feedback-regulated, which makes *sat2* rather than OASTL the stronger candidate for allelic variation affecting grain protein.

In bread wheat this locus has not been tested. We hypothesised that the *sat2* haplotype associated with a higher protein content in durum wheat has the same direction of effect in bread winter wheat. The objectives were (i) to estimate the effect of the haplotype on protein content and eleven further traits across 16 year × management environments of five harvest years; (ii) to test whether the effect depends on the harvest year and on the crop management level; (iii) to test whether the association is explained by cultivar origin, by *Rht-B1*, by the glutenin loci *Glu-A1* and *Glu-D1* and the *1BL.1RS* translocation, or by yield dilution; and (iv) to locate the exonic substitution in the predicted structure of the *SAT2* protein.

## 2. Results

### 2.1. Association of sat2 with Grain Protein Content

In the full data set (5913 observations, 65 cultivars, 16 year × management environments at one site) Hap1 exceeded Hap2 in protein content: +0.576%, SE 0.176, 95% CI 0.22–0.93, df = 61.8, *p* = 0.0017 (Figure 1, Table 1); after correction for multiplicity within the family of near-infrared (NIR)-derived indices (eight traits), q = 0.0139. Wet gluten content (+1.150%, q = 0.0296) and Zeleny sedimentation (+5.06 mL, q = 0.0296) differed in the same direction, but both are computed by the instrument from the same spectrum as protein content and are not independent confirmations (Section 2.2). The traits measured independently of NIR analysis did not differ: grain yield −1.76 dt/ha (*p* = 0.284), thousand-kernel weight +1.16 g (*p* = 0.155), test weight −0.56 g/L (*p* = 0.856) and falling number +15.0 s (*p* = 0.355); q ≥ 0.47 for all four. The remaining NIR-derived indices did not differ either (*p* = 0.16–0.42; Table 1).

The two markers were completely concordant in the 65 cultivars (D′ = 1, r^2^ = 1; Table S1), so Hap1 and Hap2 coincide with the two alleles of each marker, and a model fitted to either marker alone is identical to the haplotype model. The locus accounts for 14.2% of the between-cultivar variance in protein content at an intraclass correlation of 0.64; for grain yield its contribution is negligible (0.2%; Table S2).

### 2.2. Wet Gluten and Zeleny Sedimentation Are Computed from the Same Spectrum as Protein

Wet gluten content and Zeleny sedimentation were not measured independently: the analyser computes them from the near-infrared spectrum from which protein content is obtained. The within-year coefficient of determination of wet gluten on protein content was 0.83–0.98, the regression slope shifted between years from 1.80 to 2.83 with changes of the calibration equation, and the ratio of the gluten effect to the protein effect within each year (1.84–2.66) followed this slope; for Zeleny sedimentation R^2^ was 0.87 (2021) and 0.89 (2022) (Figure 2, Table 2). The differences between haplotypes in these two indices therefore follow from the difference in protein content.

### 2.3. The Effect Size Depends on the Year

The effect was present in all five harvest years with the same sign but differed in magnitude: the *sat2* × year interaction for protein content is significant (F = 4.52, *p* = 0.0013; q = 0.016 with correction across twelve traits). The effect was largest in 2021, the year with the highest trial mean protein content (15.9%) and the lowest precipitation: +0.937% (*p* = 0.0015). In the other years the estimates were +0.492% (2022), +0.323% (2023), +0.430% (2024) and +0.490% (2025, *p* = 0.023). In 2022–2024, with 41–43 cultivars in the yearly subsamples, the estimates did not reach significance; the minimum detectable effects of these subsamples (0.53–0.73%) are given in Table 3 (Figure 3).

A *sat2* × year interaction also passed the multiplicity correction for starch content (*p* = 0.0041; q = 0.024) and grain yield (*p* = 0.0096; q = 0.038). Neither trait showed a main effect of the haplotype, so these interactions reflect a year-to-year fluctuation of estimates around zero.

### 2.4. No Interaction with the Crop Management Level Was Detected

No interaction between the haplotype and the crop management level was detected. Across the three levels applied in all five harvest years (T1–T3; 15 environments) *p* = 0.283 for protein content, 0.152 for wet gluten content, 0.823 for Zeleny sedimentation and 0.867 for grain yield; none of the interactions with crop management pass the multiplicity correction (q ≥ 0.75; Table 4). Because a non-significant interaction does not establish equivalence, the simple effects within levels were estimated directly (Table S3): the protein content was +0.55% (95% CI 0.18–0.92) under T1, +0.49% (0.12–0.86) under T2 and +0.66% (0.29–1.03) under T3; the difference T3 − T1 was +0.11% (95% CI −0.10 to +0.33; minimum detectable difference 0.30%). Under T4, applied in 2021 only, the effect was +0.84% (0.25–1.43). The data are thus compatible with differences in the haplotype effect between the extreme levels of up to about 0.3% of protein content. Intensification itself raised grain yield under T3 relative to T1 by 8.7–20.2 dt/ha in every year, whereas its effect on protein content ranged from +0.06% (2022) to +2.26% (2024) (Table S4).

### 2.5. Cultivar Origin and the Glutenin and 1BL.1RS Loci

Haplotype frequencies were unrelated to the recorded origin group (χ^2^ = 4.28, df = 2, permutation *p* = 0.133) and to the breeding centre (permutation *p* = 0.54; Table S5), and the origin covariate left the estimate unchanged (+0.569%, *p* = 0.0037). Genome-wide marker data are not available for the collection, so population structure could not be controlled by a kinship matrix or principal components. An exploratory comparison showed a larger effect in cultivars of central-zone breeding (+0.944%, *p* = 0.00052, 23 cultivars) than of southern breeding (+0.419%, *p* = 0.19, 26 cultivars; minimum detectable effect 0.86%); the *sat2* × origin interaction is not significant (F = 2.10, *p* = 0.15). Within centres the effect is significant in cultivars of Shchelkovo Agrokhim (+0.881%, *p* = 0.0016, 13 cultivars) and ANC Donskoy (+1.016%, *p* = 0.027, 14 cultivars) and absent in cultivars of the Lukyanenko centre (−0.188%, *p* = 0.65, 12 cultivars); these subsamples are small and the comparisons exploratory (Figure 4, Table S6).

Three further quality loci genotyped in the collection were added as covariates: *Glu-A1* (three alleles, 47 cultivars with an unambiguous call), *Glu-D1* (two alleles, 45 cultivars) and the *1BL.1RS* translocation (55 cultivars); *Glu-B1* was nearly monomorphic (46 of 48 cultivars) and *Gpc-B1* was not genotyped. Haplotype frequencies were not associated with these loci (Fisher’s exact test *p* = 0.08–0.88). On the cultivars typed for each locus the protein estimate changed from +0.86% to +0.71% with *Glu-A1* (*p* = 0.0014), from +0.79 to +0.76% with *Glu-D1* (*p* = 0.0011) and from +0.70 to +0.69% with *1BL.1RS* (*p* = 0.0010); with all three covariates it changed from +0.84 to +0.71% (42 cultivars; *p* = 0.0039). Part of the effect is thus shared with *Glu-A1*, whose null allele is more frequent among Hap2 cultivars (six of 33 against one of 32). When the heterogeneous *Glu-A1* and *Glu-D1* samples are entered as separate factor levels instead of being treated as missing, the adjusted estimates are +0.47% (*Glu-A1* and *1BL.1RS*; 53 cultivars; *p* = 0.018) and +0.56% (all four loci; 47 cultivars; *p* = 0.015). The estimates without covariates on these subsamples exceed the whole-panel estimate because the cultivars lacking glutenin calls are not a random subset (Table S7).

### 2.6. Rht-B1 and Grain Yield as Covariates

*Rht-B1* was typed for 60 cultivars (27 with the dwarfing alleles *Rht-B1b* or *Rht-B1e*, 33 with the wild-type *Rht-B1a*); its allelic state was distributed independently of the *sat2* haplotype (χ^2^ = 0.000, permutation *p* = 1.00). On the 59 cultivars typed for both loci the effect is +0.491% (*p* = 0.0074) without and +0.490% (*p* = 0.0079) with the covariate, and no *sat2* × Rht-B1 interaction was detected (*p* = 0.45 for protein content; Table S8); the difference from the whole-panel estimate reflects the composition of the subsample. With grain yield as a linear covariate the effect of Hap1 persists: +0.534% (SE 0.155; *p* = 0.0010); the yield coefficient is −0.022% protein per dt/ha, so a dilution effect is present in the data. The covariate entered uncentred, so the contrast is estimated at the mean yield of the data set (86.3 dt/ha); centring it changes only the intercept. The advantage of Hap1 is therefore retained at an equal yield level.

### 2.7. Structural Context of the Gly325Ser Substitution

The substitution was located in the AlphaFold DB model of the subgenome B homoeolog of *sat2* in bread wheat (UniProt A0A3B6IYA1, gene TraesCS4B02G330300, 338 residues), the chromosome 4B copy on which the polymorphism was identified in durum wheat [17]; the durum wheat entry of equal length (A0A9R0WG02) differs from it only at position 325 (Gly versus Ser), so both allelic states occur in the reference sequences of the two species. *Gly325Ser* lies at the C-terminal edge of the final helix (residues 317–326), thirteen residues from the C-terminus, in a region predicted with low confidence (pLDDT ≈ 50 at residue 325; 30–46 in the following disordered segment). The catalytic core (residues 61–313) is predicted with confidence (pLDDT > 70) and coincides in the models of the two allelic states to within 0.16 Å over Cα atoms, so no effect on the core is predicted (Figure 5).

AlphaFold is not intended for evaluating single substitutions, so the local geometry is an illustration rather than evidence of a functional change. In the model the side chain of *Ser325* is predicted to hydrogen bond the carbonyls of *Glu321* and *His322*, capping the final turn of the helix; glycine cannot form this cap and leaves the following segment mobile. The C-terminal region of plant serine acetyltransferase participates in the formation of the cysteine synthase complex with OASTL [18,19,20], so a change in its mobility could shift the assembly equilibrium and the supply of cysteine to the developing grain; this remains a hypothesis that requires measurement of enzyme activity and complex formation for the two allelic forms. Among nineteen grass orthologs with more than 70% identity, position 325 is occupied by glycine in five sequences and by serine in two; in the panicoid grasses, whose C-terminal sequence differs, it is hydrophobic (Table S9). The position is not strictly conserved, which is compatible with the moderate magnitude of the effect.

## 3. Discussion

This is the first evidence that the *sat2* polymorphism described as a candidate for protein content in durum wheat [17] is associated with grain protein content in bread winter wheat. Hap1, which carries G at TDsat2.e9.1 (*Gly325*) and T at TDsat2.i1.1, exceeded Hap2, the haplotype of the Chinese Spring reference genome, by 0.58% across 16 year × management environments of five harvest years at one site. The direction agrees with durum wheat, where the higher protein content is associated with nucleotide G of exon 9 [17]. Haplotype frequencies were unrelated to recorded cultivar origin and to *Rht-B1*, the effect persisted after adjustment for *Glu-A1*, *Glu-D1*, *1BL.1RS*, and grain yield, and no interaction with the crop management level was detected within the power of the study.

The markers are not anonymous tags. Both polymorphisms lie inside *sat2* on chromosome 4B, in subgenome B, which is shared by durum (AABB) and bread wheat (AABBDD), so they type the same homoeolog. The effect of the exonic variant was confirmed in durum wheat in recombinant inbred lines, where population structure is excluded by design, whereas the intronic variant rests on association evidence alone [17]. Together with the location of *Gly325Ser* in the regulatory C-terminal region of the enzyme, this provides a plausible functional hypothesis, not a demonstration of causality. The smaller effect in bread wheat (+0.58% against +0.92–1.33% in durum wheat [17]) may reflect homoeologous redundancy, since bread wheat carries a third copy of the gene in subgenome D, and the different genetic background of Russian winter cultivars.

The effect kept its sign in all five years but was about twice as large in 2021, the year with the highest trial mean protein content. Differences in the efficiency of sulphur use may become visible when protein accumulation is not constrained by other factors; with five harvest years and covarying weather variables this cannot be tested here and requires trials with a controlled sulphur supply.

For breeding, the haplotype can serve as an additional selection criterion in any year, most usefully in the pre-selection of parents and early-generation lines. The gain may be larger under conditions favourable for protein accumulation, but restricting selection to such environments is not warranted, because the differences between management levels are bounded by the data only to about ±0.3% (Section 2.4). Single-locus markers of this kind complement rather than replace genomic prediction and other data-driven approaches to crop breeding [21,22]; a locus with a confirmed effect, such as the *sat2* haplotype, can enter genomic-prediction models as a fixed covariate, which would also test its contribution in broader germplasm. Because the effect was not detected in the cultivars of one breeding centre (Section 2.5), the association should be checked within a programme before the marker is applied to its material.

Whether the *sat2* haplotype affects gluten quality itself, through the availability of cysteine for disulfide bond formation, remains open: in the present data wet gluten content and Zeleny sedimentation are computed from the same spectrum as protein content, and the question requires gluten washing, rheological tests and measurement of the cysteine content of the grain.

### Limitations

The following limitations apply to this study.

- Wet gluten content, Zeleny sedimentation, starch content, vitreousness and the contents of crude fat, crude fibre and phosphorus are computed by the near-infrared analyser from a single spectrum and are not independent measurements; protein content is the output of the manufacturer’s calibration, whose reference method and accuracy are not available to the authors. Grain yield, thousand-kernel weight, test weight and falling number were measured independently.
- All cultivars were grown in a single trial at one site, so the 16 environments are year × management combinations at that site; the harvested plot area differed between years (17.3–41.1 m^2^) and between management levels within a year. Extrapolation to other regions requires multi-site trials.
- The minimum detectable effect for protein content at 80% power is 0.49%; equivalence of the haplotype effect across management levels was not established, the data bounding the difference between the extreme levels only to about ±0.3% of protein content (Table S3).
- Genome-wide marker data are not available for the collection, so population structure and relatedness could not be controlled by a kinship matrix or principal components; *Glu-A1*, *Glu-D1* and *1BL.1RS* were included as covariates only for the 42–55 cultivars typed for them, *Gpc-B1* was not genotyped, and the breeding-centre subsamples comprise 12–14 cultivars with protein records.
- The nucleotide identity of the two variants is assigned from allele-specific PCR with primers designed on the gene sequenced in durum wheat [17]; the bread wheat cultivars studied here were not sequenced.
- The association was tested in a single collection of Russian bread winter wheat cultivars; confirmation in collections of different origin and genetic background is required before the marker is recommended beyond this gene pool.

## 4. Materials and Methods

### 4.1. Plant Material

The material was a germplasm panel of 65 registered cultivars and advanced breeding lines of bread winter wheat (*Triticum aestivum* L.) of different origin, maintained by Shchelkovo Agrokhim JSC for breeding purposes and not assembled for the present study (Table S5). It is not a mapping population: the accessions are independent cultivars from four Russian breeding centres and from foreign breeding, and no pedigree relationships beyond those recorded in Table S5 are known. Both *sat2* markers were applied to the whole collection, and the analysis comprises the 65 cultivars with field data of the 2021–2025 harvests that carry one of the two frequent haplotypes (Section 4.2); the two cultivars carrying the rare third haplotype were excluded because no effect can be estimated from two carriers. High-molecular-weight DNA was extracted from four-day-old seedlings in three replicates by the CTAB method [23].

### 4.2. Genotyping

The allelic state of the two *sat2* polymorphisms was determined by Kompetitive Allele-Specific PCR (KASP-PCR) with two markers developed at the All-Russian Research Institute of Agricultural Biotechnology (VNIISB) [17] for defined nucleotide polymorphisms of the gene (Table 5). The gene was identified in durum wheat as a candidate from a genome-wide association study and sequenced, so the polymorphisms are known at the sequence level: TDsat2.e9.1 types a G>A substitution in exon 9 that results in the amino-acid substitution *Gly325Ser* (nucleotide G corresponds to Gly325), and TDsat2.i1.1 a G>T substitution in intron 1 [17]. Both markers are intragenic and discriminate known nucleotide variants rather than anonymous polymorphisms.

Haplotype classification was performed as follows. Each accession was assigned to one of the four possible two-marker haplotypes defined by the nucleotides at TDsat2.e9.1 and TDsat2.i1.1 (Table 6). In the 65 analysed cultivars the two markers were completely concordant: 32 cultivars carry Hap1 (G; T) and 33 carry Hap2 (A; G); Hap3 (G; G) was found in two cultivars of the collection (Zodiak and Lyutescens 982). With only two carriers no estimate of a haplotype effect is possible, so these cultivars were excluded from all models although their field records are available, and Hap4 (A; T) was not observed (D′ = 1 and r^2^ = 1 in the 65 cultivars; r^2^ = 0.89 over the 67 accessions with calls; Table S1). The bread wheat reference genome (cv. Chinese Spring, IWGSC RefSeq v1.0, gene TraesCS4B02G330300) carries A at TDsat2.e9.1 and G at TDsat2.i1.1, that is, Hap2; this was established by locating the allele-specific primers of Table 5 in the reference gene sequence. The haplotype and the call at each marker are given for every cultivar in Table S5.

Reactions were run in 96-well plates. A 10 µL mixture contained 5 µL of Master Mix KBS-1050-102 (LGC Biosearch Technologies, Hoddesdon, UK) with FAM, HEX and ROX dyes, the allele-specific and common primers (0.15 µM of each allele-specific primer and 0.375 µM of the common primer) and 5 µL of DNA (50 ng). Amplification was performed as follows: 94 °C for 15 min; 10 cycles of 94 °C for 20 s and 61 °C for 60 s with the annealing temperature decreasing by 0.6 °C per cycle; 35 cycles of 94 °C for 20 s and 55 °C for 60 s; and the signal was read at 37 °C for 60 s. Data were processed in Bio-Rad CFX Manager 3.1.

### 4.3. Field Trials

Trials were conducted in Oryol Oblast (52.9319° N, 36.0631° E, 194 m a.s.l.) on leached chernozem by NPO Betagran Seeds LLC as competitive variety trials of cultivars and breeding lines with check cultivars (Moskovskaya 39, Skipetr, Grom). Plots were sown with a Klin-1.5 drill (sown area 63 m^2^, 3 × 21 m; 15 cm row spacing; 3–5 cm seeding depth). The harvested plot area was smaller than the sown area and not constant: it ranged from 17.3 to 41.1 m^2^ between years and differed between management levels within a year (Table S10); yields were converted to decitonnes per hectare at 14% moisture in the primary records. The plot layout, the randomisation procedure and the positions of individual cultivars in different years were not recorded in the primary trial documentation.

The trials compared levels of crop management designated by the trial operator as technologies T1 (traditional), T2 (optimal-decision) and T3 (maximum-potential, the high-yield technology) in all five harvest years and, in 2021 only, a fourth level, T4, an integrated scheme intended to realise the genetic potential of the plants as fully as possible. According to the trial operator, the fertiliser scheme of T1–T3 was the same in all harvest years. The common background was a complex NPK(S) fertiliser (N5:P17:K36:S2) at 700 kg/ha. The differentiating applications were as follows: in autumn, ammonium sulphate (150 kg/ha) and, at T3 only, liquid complex fertiliser 11–37 (100 kg/ha); in early spring, ammonium nitrate (150 kg/ha) and UAN-32 (150 kg/ha) at all levels, with ammonium sulphate at T3 only; and at flowering, liquid complex fertiliser 11–37 (100 kg/ha at T3 and 50 kg/ha at T2) and magnesium sulphate (10 kg/ha, twice, at T3 and T2). Urea was applied at 20–30 kg/ha by growth stage and three times at T3 (10 + 20 + 20 kg/ha). T1 included plant protection products; T2 added foliar feeding with the preparations ‘Ultramag’ and ‘Biostim’ and fertilisers applied in dissolved form; T3 had an increased load of plant protection products and three foliar treatments. The 16 environments of the analysis are the combinations of harvest year and level (T1–T4 in 2021 and T1–T3 in each of 2022–2025) at this single site.

Grain yield (dt/ha), the contents of protein, wet gluten and starch, thousand-kernel weight, test weight, vitreousness, falling number and Zeleny sedimentation were recorded; the contents of crude fat, crude fibre and phosphorus were recorded in 2023–2025. The set of traits differed between years (Table S11); the analysis included 5913 observations. Growing seasons differed markedly (Table S11, Figure S1): the sum of active temperatures (>10 °C) ranged from 2343 °C (2021/22) to 3076 °C (2023/24) and annual precipitation from 517 mm (2020/21) to 779 mm (2023/24). The highest trial mean protein content (15.9%) was obtained in 2021, the year with the lowest precipitation.

### 4.4. Grain Quality Determination

The contents of protein, wet gluten, starch, vitreousness, crude fat, crude fibre and phosphorus and Zeleny sedimentation were taken from the near-infrared (NIR) whole-grain analyser InfraLum FT-12 (Lyumeks, Saint Petersburg, Russia), operated according to the manufacturer’s instructions with its standard calibration; each sample was measured in triplicate and the mean was used. Protein content is the directly calibrated variable, whereas wet gluten content and Zeleny sedimentation are computed by the instrument from the same spectrum (Section 2.2). Manual gluten washing and gluten deformation index measurements, which the laboratory uses as referee methods, were not recorded per plot in the primary record sheets and were not analysed. Details of the reference method underlying the manufacturer’s calibration and its accuracy are not available to the authors.

Grain yield was determined by weighing the grain from the harvested plot and converting it to a 14% moisture basis. Falling number was determined with ChP-3 and ChP-7 instruments (Biophysical Apparatus, Moscow, Russia) and test weight with a Purke PKh-1MTs tester (Priborinform, Rostov-on-Don, Russia), both according to the Russian state standards (GOST); thousand-kernel weight was determined by counting and weighing. These four traits were measured independently of NIR analysis.

### 4.5. Statistical Analysis

The *sat2* polymorphism was the single a priori hypothesis of the work: the collection was typed with two markers of this gene, no locus was selected on the basis of the results, and the direction of the effect was specified from the durum wheat study [17] before the bread wheat data were analysed. Rht-B1 and the glutenin loci were added only as controls for possible collinearity. The composition of the collection was determined by the cultivars available at the partner breeding company and not by any trait or marker.

Computations were performed in R 4.5.3 (packages lme4, lmerTest, emmeans); the five-year test of the haplotype × crop management interaction was computed in R 4.6.0, which reproduces the main model exactly. The cultivar was the unit of replication for the genetic effect, because the haplotype is a property of the cultivar rather than of the plot. The main model was trait ~ *sat2* haplotype + environment + (1 | cultivar), where the environment is the combination of harvest year and crop management level at the single site (16 levels); degrees of freedom were obtained by the Kenward–Roger approximation. Multiplicity was controlled by the Benjamini–Hochberg procedure separately within two trait families, eight NIR-derived indices and four independently measured traits; the interactions (*sat2* × year, *sat2* × crop management level T1–T3, *sat2* × origin, *sat2* × *Rht-B1*) were tested with the same correction within each interaction type. Because a non-significant interaction does not establish equivalence, the simple effects within each management level and the difference T3 − T1 were estimated with 95% confidence intervals and minimum detectable differences (Table S3). Additional models included the covariates of cultivar origin, *Rht-B1*, *Glu-A1*, *Glu-D1*, and *1BL.1RS* (Tables S7 and S8), grain yield as an uncentred linear covariate, and the effect within individual years. The minimum detectable effect at 80% power and α = 0.05 was computed as 2.802 × SE of the contrast and is reported for every subsample. The association of haplotype frequencies with the origin group and with Rht-B1 was tested by a χ^2^ criterion with a permutation estimate of significance (20 000 permutations) and with the glutenin and *1BL.1RS* loci by Fisher’s exact test.

The analysed data set comprised 65 cultivars for which both phenotypic data and a defined haplotype were available. Because the analysed data set contains one value per cultivar, environment and trait, within-trial error was estimated from the residual variance of the mixed model (Table S12). Quality data for 2023–2025 were taken from the primary record sheets. The full model outputs are given in Tables S1–S4, S6–S8 and S12–S14.

## 5. Conclusions

1. Hap1 of *sat2* (G at TDsat2.e9.1, Gly325; T at TDsat2.i1.1) exceeds Hap2 (A; G, the haplotype of the Chinese Spring reference genome) in grain protein content by 0.58% (95% CI 0.22–0.93; *p* = 0.0017; q = 0.014) across 16 year × management environments of five harvest years at one site. This is the first evidence of the association in *Triticum aestivum* L., and its direction agrees with durum wheat.
2. The effect is not explained by yield dilution, by *Rht-B1* or by recorded cultivar origin and persists (+0.71%) after adjustment for *Glu-A1*, *Glu-D1* and the *1BL.1RS* translocation; grain yield and the other independently measured traits do not differ between haplotypes.
3. The effect kept its sign in all five years but was largest in the year most favourable for protein accumulation. No haplotype × crop management interaction was detected across levels T1–T3 of five harvest years; the data bound the difference between extreme levels to about ±0.3% of protein content.
4. The *Gly325Ser* substitution lies in the regulatory C-terminal region of *SAT2*, which provides a plausible functional hypothesis for the association. Confirmation in collections of different origin and genetic background is required before the markers are recommended for marker-assisted selection beyond this gene pool.

## Figures and Tables

**Figure 1:**
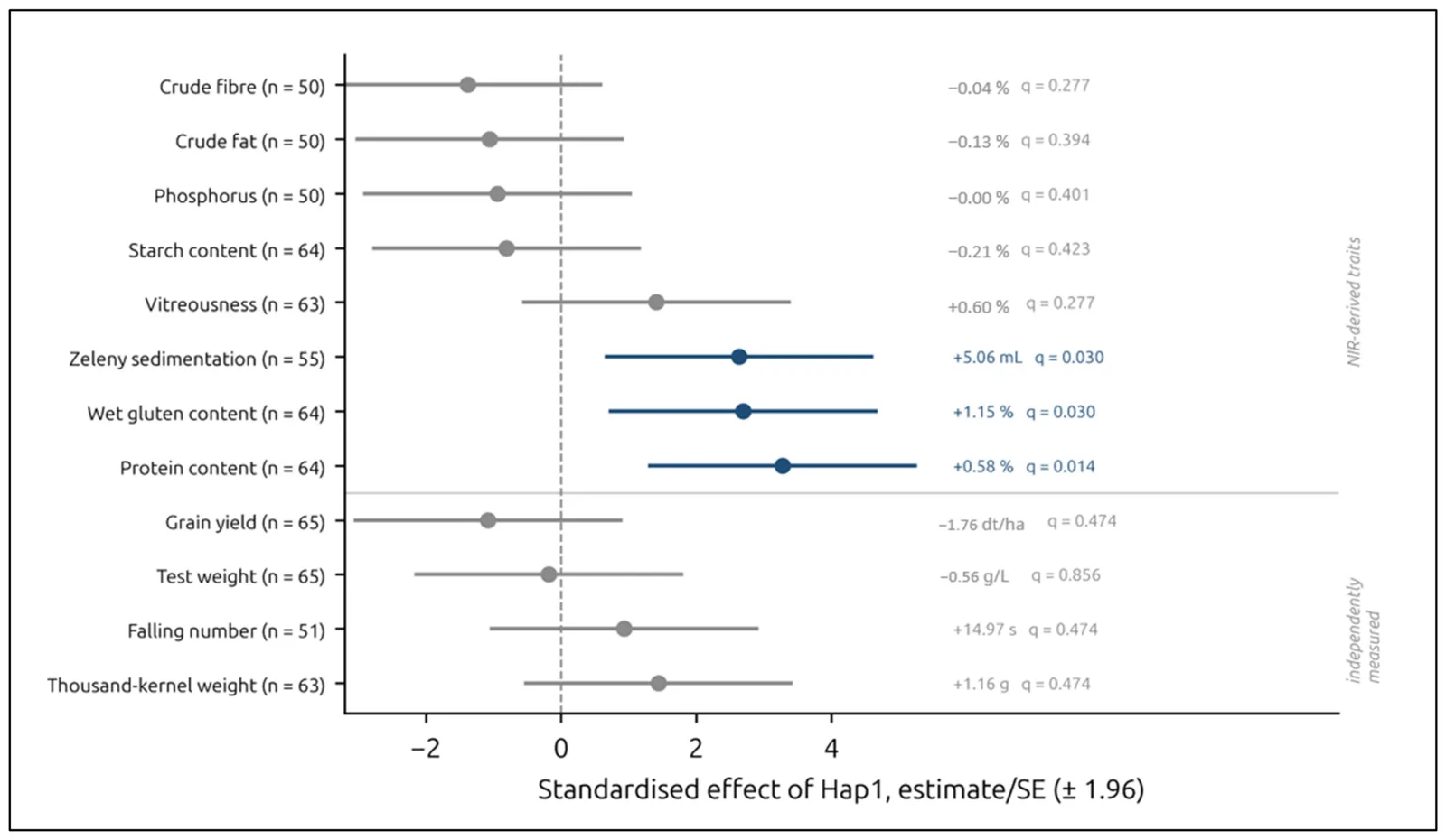
Effect of the *sat2* haplotype (contrast Hap1 − Hap2) on twelve quality and productivity traits (65 cultivars, 16 environments formed by five harvest years and the crop management levels T1–T4 (2021) or T1–T3 (2022–2025) at one site). Points are standardised effects (estimate/SE) with ±1.96 intervals; the number of cultivars with data for each trait is given in the axis labels, and the estimate in trait units with the q value is printed on the right. The horizontal line separates indices computed by the near-infrared analyser (upper group) from independently measured traits (lower group); the Benjamini–Hochberg correction was applied separately within each group. Blue, q < 0.05; grey, q ≥ 0.05.

**Figure 2:**
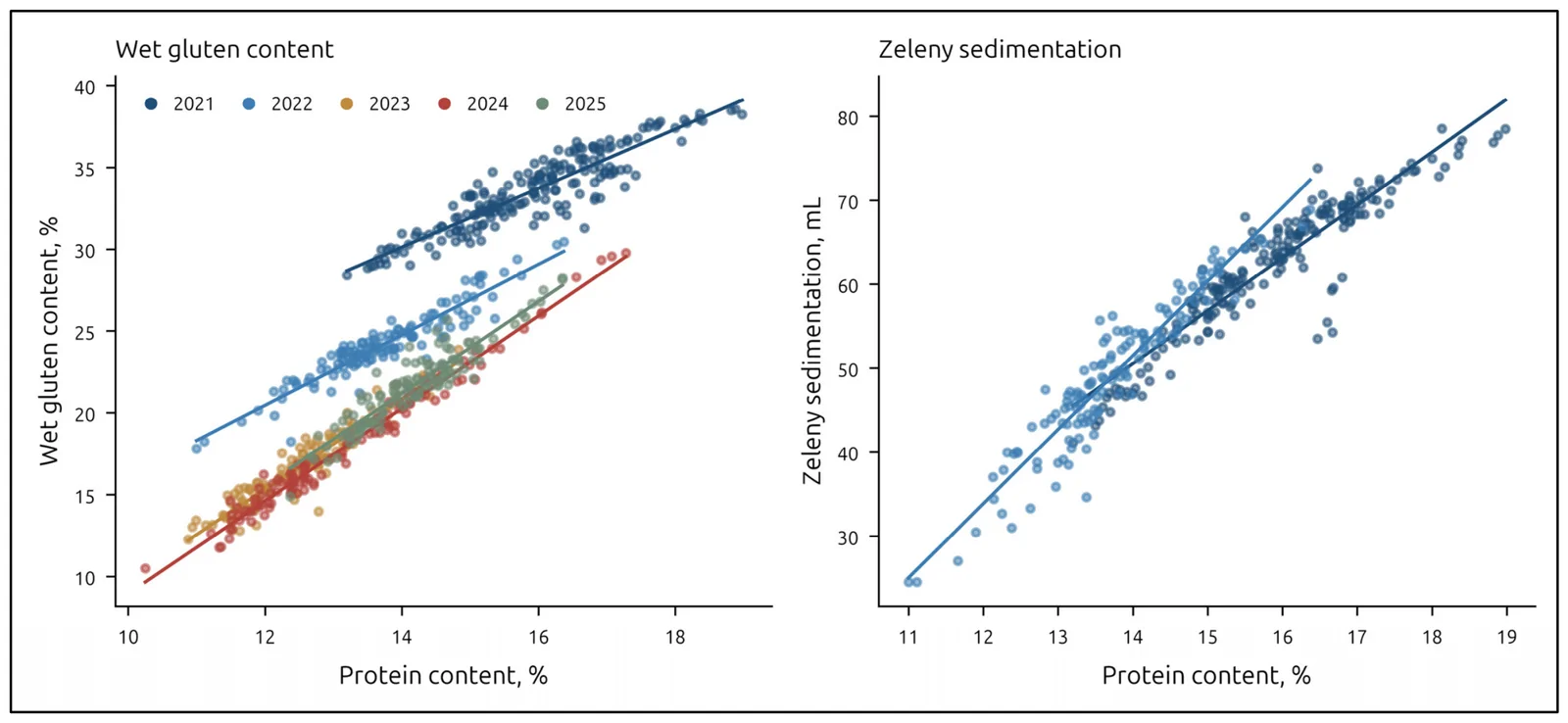
Relationship of wet gluten content (**left**) and Zeleny sedimentation (**right**) to protein content within each harvest year. Each point is one cultivar in one environment; lines are within-year regressions, coloured by harvest year. The slope of wet gluten on protein content shifts between years from 1.80 to 2.83 with changes in the calibration equation of the instrument; coefficients of determination are given in Table 2. Zeleny sedimentation was recorded in 2021 and 2022 only.

**Figure 3:**
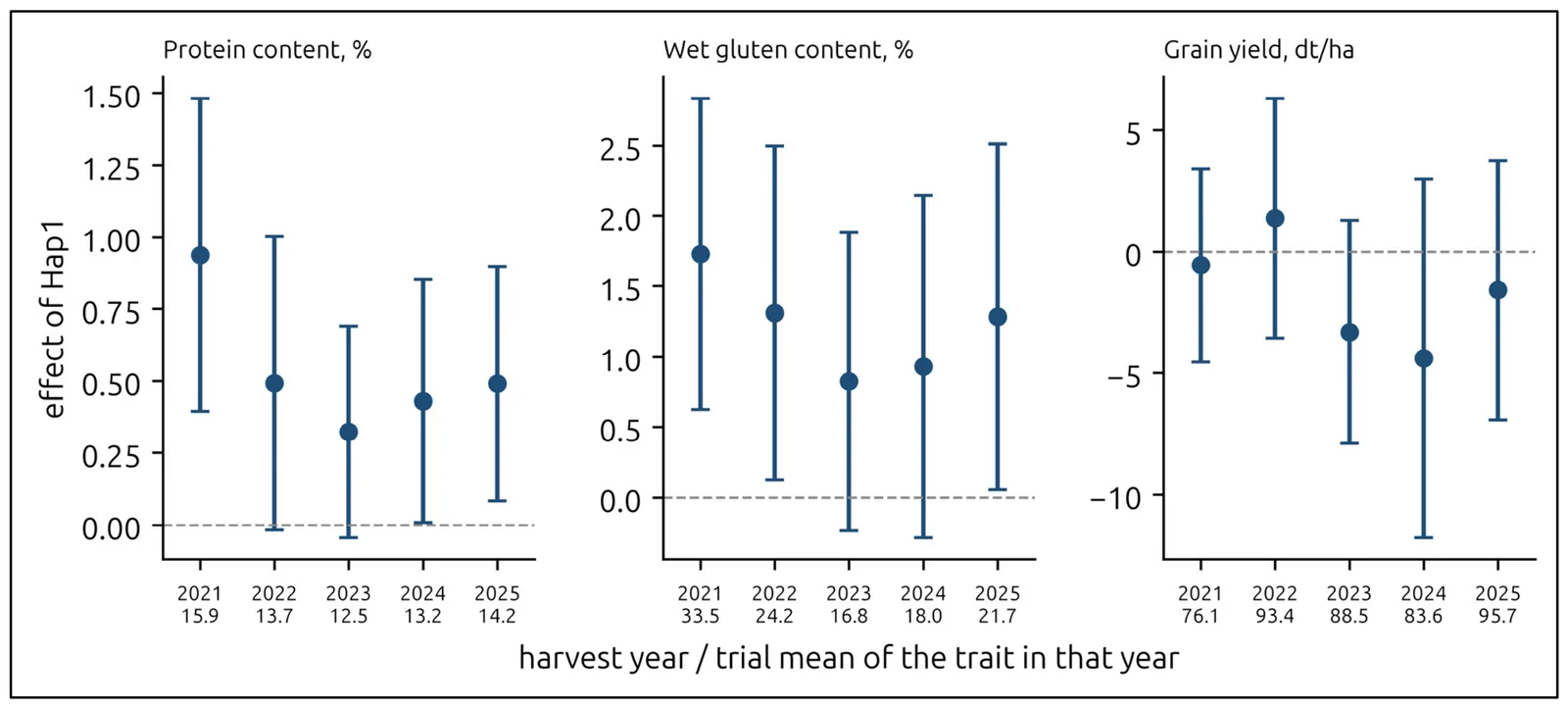
Effect of Hap1 within individual harvest years for protein content, wet gluten content and grain yield. Points are the within-year contrast Hap1 − Hap2 with ± 95% confidence intervals; below the axis, the harvest year and the trial mean of the trait in that year. The sign of the protein effect is the same in all five years (*sat2* × year: *p* = 0.0013), and grain yield did not differ between haplotypes in any year. Wet gluten content is computed by the near-infrared analyser from the same spectrum as protein content.

**Figure 4:**
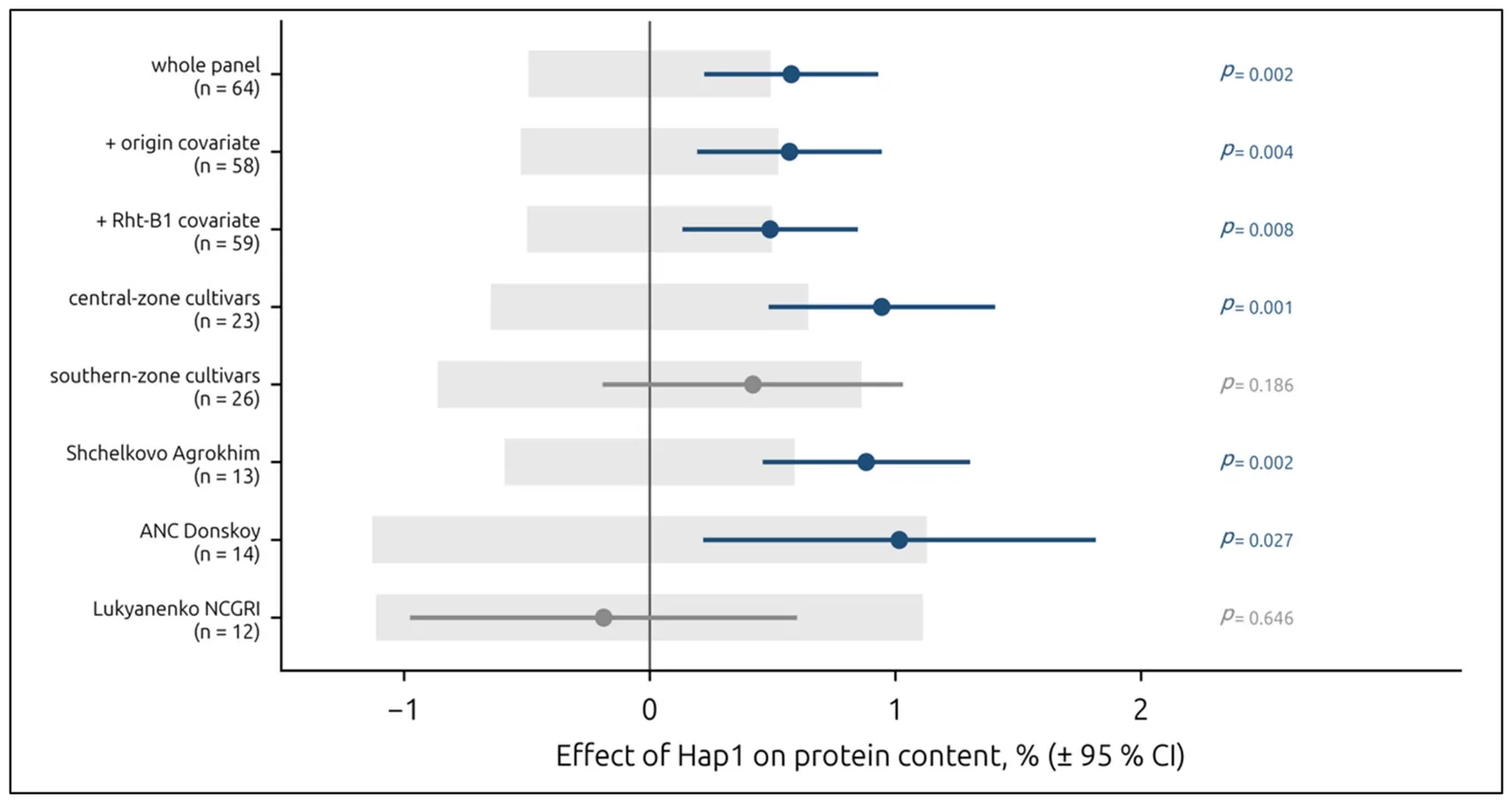
Effect of Hap1 on protein content with the origin and *Rht-B1* covariates and in subsamples by origin zone and breeding centre. Points are estimates with ± 95% confidence intervals; the grey band is the minimum detectable effect of the subsample at 80% power and α = 0.05: an estimate inside the band could not be distinguished from zero at that number of cultivars. Blue, *p* < 0.05; grey, *p* ≥ 0.05. The haplotype × origin interaction is not significant (*p* = 0.15); the breeding-centre subsamples comprise 12–14 cultivars with protein records.

**Figure 5:**
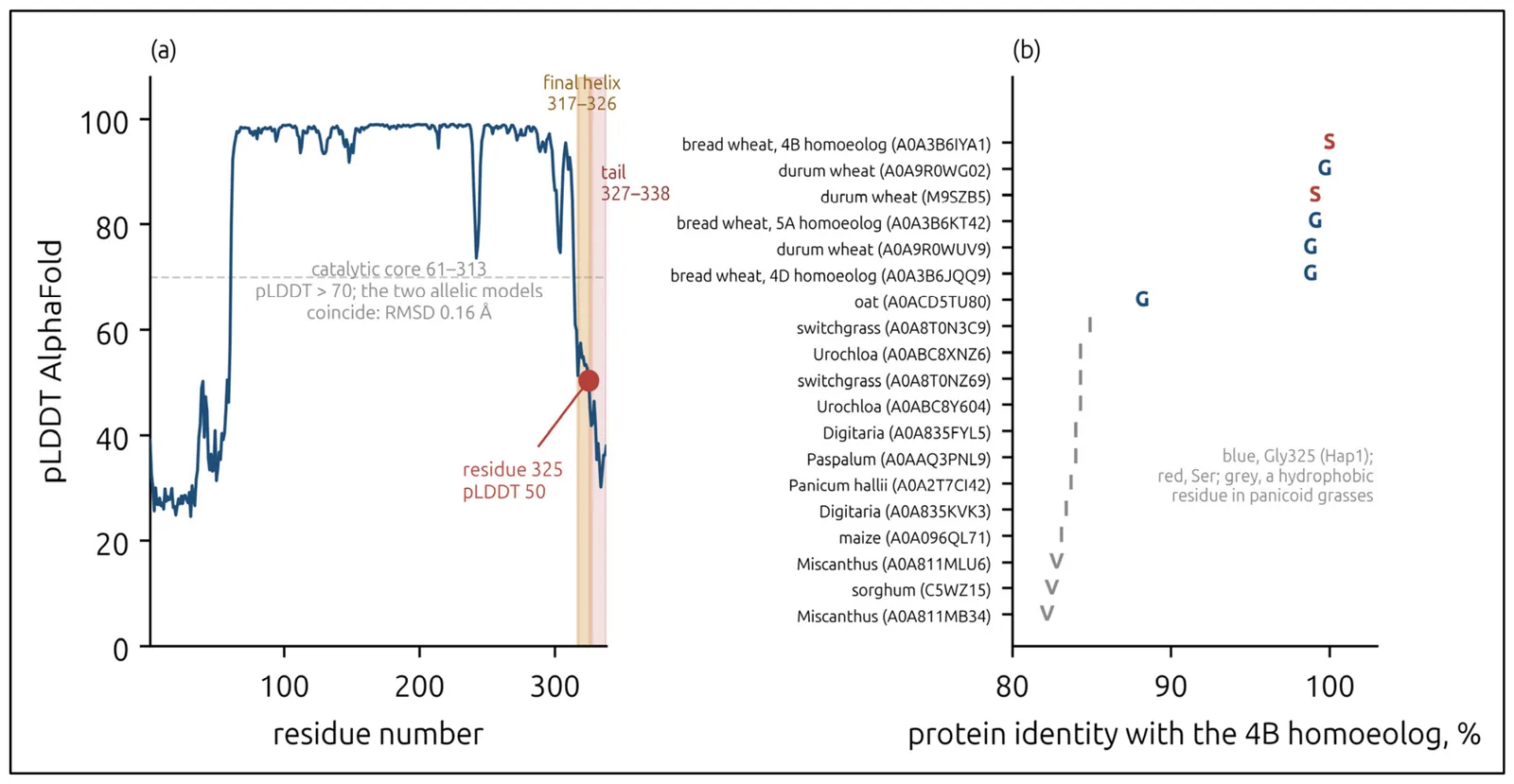
Structural context of the Gly325Ser substitution in the *SAT2* protein, based on the AlphaFold DB model of the bread wheat 4B homoeolog (UniProt A0A3B6IYA1, gene TraesCS4B02G330300, 338 residues). (**a**) Prediction confidence (pLDDT) along the chain; the shaded bands mark the final helix (residues 317–326, orange) and the disordered C-terminal segment (327–338, red), and the dashed line marks pLDDT = 70. The predicted hydrogen bonds of *Ser325* to the carbonyls of *Glu321* (2.72 Å) and *His322* (2.97 Å) are putative, as the local confidence at this residue is low (pLDDT ≈ 50). (**b**) Amino acid residue at the equivalent position in grass orthologs with more than 70% identity to the 4B homoeolog: blue, *Gly325* (Hap1); red, Ser; grey, a hydrophobic residue in panicoid grasses, whose C-terminal sequence differs.

**Table 1:** Effect of the *sat2* haplotype (contrast Hap1 − Hap2) in the full data set (2021–2025, 16 year × management environments at one site). MDE (80%), minimum detectable effect at 80% power and α = 0.05, computed as 2.802 × SE of the contrast. Protein content is recorded for 64 of the 65 cultivars.

Trait	Family	Obs.	Cultivars	Cultivar Hap1	Cultivar Hap2	Estimate	SE	df	*p*	q (FDR)	MDE (80%)
Protein content, %	NIR-derived	682	64	32	32	0.576	0.176	61.8	0.0017	0.014	0.493
Wet gluten content, %	NIR-derived	680	64	32	32	1.150	0.427	61.8	0.0091	0.030	1.197
Zeleny sedimentation, mL	NIR-derived	321	55	29	26	5.063	1.923	52.9	0.0111	0.030	5.389
Thousand-kernel weight, g	independently measured	436	63	32	31	1.163	0.808	60.5	0.1552	0.474	2.264
Vitreousness, %	NIR-derived	534	63	32	31	0.604	0.429	60.6	0.1642	0.277	1.202
Crude fibre, %	NIR-derived	358	50	26	24	−0.039	0.028	47.6	0.1733	0.277	0.079
Grain yield, dt/ha	independently measured	698	65	32	33	−1.757	1.626	62.4	0.2841	0.474	4.556
Crude fat, %	NIR-derived	324	50	26	24	−0.127	0.120	45.9	0.2959	0.394	0.337
Phosphorus, %	NIR-derived	358	50	26	24	−0.003	0.003	46.9	0.3511	0.401	0.009
Falling number, s	independently measured	273	51	26	25	14.971	16.047	48.9	0.3554	0.474	44.963
Starch content, %	NIR-derived	681	64	32	32	−0.207	0.256	61.2	0.423	0.423	0.719
Test weight, g/L	independently measured	568	65	32	33	−0.561	3.083	61.4	0.8561	0.856	8.638

**Table 2:** Relationship of the NIR-derived quality indices to protein content within a harvest year.

Harvest Year	Trait	Observations	R^2^	Regression Slope
2021	Wet gluten content	193	0.833	1.80
2022	Wet gluten content	129	0.886	2.15
2023	Wet gluten content	123	0.903	2.76
2024	Wet gluten content	115	0.978	2.83
2025	Wet gluten content	120	0.872	2.81
2021	Zeleny sedimentation	192	0.868	6.30
2022	Zeleny sedimentation	129	0.893	8.83

**Table 3:** Effect of Hap1 (contrast Hap1 − Hap2) within individual harvest years. MDE (80%), minimum detectable effect at 80% power and α = 0.05 (2.802 × SE of the contrast); a non-significant estimate smaller than the MDE should not be read as absence of an effect. Zeleny sedimentation was recorded in 2021 and 2022 only.

Trait	Harvest Year	Trial Mean	Obs.	Cultivars	Cultivar Hap1	Cultivar Hap2	Estimate	SE	df	*p*	MDE (80%)
Protein content	2021	15.91	195	49	26	23	0.937	0.277	47.0	0.0015	0.777
Protein content	2022	13.74	129	43	22	21	0.492	0.260	41.0	0.0652	0.728
Protein content	2023	12.54	123	41	23	18	0.323	0.187	39.0	0.0928	0.525
Protein content	2024	13.20	115	41	21	20	0.430	0.215	38.7	0.0524	0.602
Protein content	2025	14.18	120	40	21	19	0.490	0.207	38.0	0.0233	0.581
Wet gluten content	2021	33.52	193	49	26	23	1.729	0.564	47.0	0.0036	1.580
Wet gluten content	2022	24.19	129	43	22	21	1.310	0.607	41.0	0.0369	1.701
Wet gluten content	2023	16.81	123	41	23	18	0.823	0.541	39.0	0.1365	1.517
Wet gluten content	2024	17.99	115	41	21	20	0.929	0.621	38.6	0.1427	1.739
Wet gluten content	2025	21.69	120	40	21	19	1.284	0.627	38.0	0.0474	1.756
Zeleny sedimentation	2021	62.40	192	49	26	23	5.144	1.976	47.0	0.0123	5.537
Zeleny sedimentation	2022	49.17	129	43	22	21	4.392	2.509	41.0	0.0875	7.031
Thousand-kernel weight	2021	41.44	172	43	23	20	0.493	1.105	41.0	0.658	3.096
Thousand-kernel weight	2023	44.94	126	42	24	18	2.269	1.379	40.0	0.1077	3.863
Thousand-kernel weight	2025	48.36	120	40	21	19	1.092	1.297	38.0	0.4052	3.635
Grain yield	2021	76.12	208	52	27	25	−0.563	2.031	50.0	0.7827	5.690
Grain yield	2022	93.38	132	44	22	22	1.368	2.513	42.0	0.589	7.042
Grain yield	2023	88.46	123	41	23	18	−3.319	2.341	39.0	0.1642	6.559
Grain yield	2024	83.58	115	41	21	20	−4.392	3.772	38.8	0.2515	10.570
Grain yield	2025	95.72	120	40	21	19	−1.595	2.724	38.0	0.5617	7.633

**Table 4:** Tests of the haplotype × environment and haplotype × collection structure interactions (Kenward–Roger F tests; q, Benjamini–Hochberg correction within each interaction type). The crop management test uses levels T1–T3 of all five harvest years. Zeleny sedimentation was recorded in two harvest years only, so its interaction with year has one degree of freedom and F ≈ 0 (within-year estimates +5.08 and +5.03 mL in the interaction model; the separate within-year models in Table 3 give +5.14 and +4.39 mL).

Trait	Interaction	df	F	*p*	q (FDR)
Protein content	*sat2* × year	614.7	4.516	0.0013	0.016
Protein content	*sat2 ×* crop management level (T1–T3, 2021–2025)	553.2	1.266	0.2827	0.848
Protein content	*sat2* × origin zone	44.9	2.101	0.1541	0.626
Protein content	*sat2* × *Rht-B1*	54.9	0.586	0.4471	0.766
Wet gluten content	*sat2* × year	613.0	2.597	0.0354	0.106
Wet gluten content	*sat2 ×* crop management level (T1–T3, 2021–2025)	552.2	1.893	0.1516	0.753
Wet gluten content	*sat2* × origin zone	44.9	1.041	0.313	0.626
Wet gluten content	*sat2* × *Rht-B1*	54.9	1.172	0.2838	0.766
Starch content	*sat2* × year	633.4	3.871	0.0041	0.024
Starch content	*sat2 ×* crop management level (T1–T3, 2021–2025)	553.3	1.675	0.1883	0.753
Starch content	*sat2* × origin zone	44.2	0.300	0.5865	0.808
Starch content	*sat2* × *Rht-B1*	54.7	0.705	0.4046	0.766
Vitreousness	*sat2* × year	486.9	1.330	0.2639	0.528
Vitreousness	*sat2* × crop management level (T1–T3, 2021–2025)	414.9	0.122	0.8856	0.956
Vitreousness	*sat2* × origin zone	45.9	2.363	0.1311	0.626
Vitreousness	*sat2* × *Rht-B1*	54.6	0.308	0.5811	0.872
Crude fat	*sat2* × year	291.4	0.836	0.4343	0.600
Crude fat	*sat2 ×* crop management level (T1–T3, 2021–2025)	268.0	0.124	0.8832	0.956
Crude fat	*sat2* × origin zone	34.9	0.495	0.4864	0.808
Crude fat	*sat2* × *Rht-B1*	42.5	0.051	0.8227	0.985
Crude fibre	*sat2* × year	315.8	0.453	0.6359	0.763
Crude fibre	*sat2* × crop management level (T1–T3, 2021–2025)	298.6	1.975	0.1405	0.753
Crude fibre	*sat2* × origin zone	35.9	0.271	0.6057	0.808
Crude fibre	*sat2* × *Rht-B1*	44.3	0.963	0.3317	0.766
Phosphorus	*sat2* × year	323.4	0.294	0.7458	0.814
Phosphorus	*sat2 ×* crop management level (T1–T3, 2021–2025)	299.5	0.308	0.7353	0.956
Phosphorus	*sat2* × origin zone	35.4	0.015	0.902	0.902
Phosphorus	*sat2* × *Rht-B1*	43.5	0.650	0.4244	0.766
Zeleny sedimentation	*sat2* × year	268.0	0.003	0.9566	0.957
Zeleny sedimentation	*sat2 ×* crop management level (T1–T3, 2021–2025)	211.1	0.195	0.8230	0.956
Zeleny sedimentation	*sat2* × origin zone	41.9	1.265	0.2672	0.626
Zeleny sedimentation	*sat2* × *Rht-B1*	46.2	0.132	0.7183	0.958
Grain yield	*sat2* × year	642.6	3.371	0.0096	0.038
Grain yield	*sat2 ×* crop management level (T1–T3, 2021–2025)	565.8	0.143	0.8672	0.956
Grain yield	*sat2* × origin zone	45.8	1.047	0.3116	0.626
Grain yield	*sat2* × *Rht-B1*	55.6	0.000	0.9854	0.985
Thousand-kernel weight	*sat2* × year	395.3	1.056	0.3679	0.600
Thousand-kernel weight	*sat2 ×* crop management level (T1–T3, 2021–2025)	317.7	0.045	0.9558	0.956
Thousand-kernel weight	*sat2* × origin zone	45.6	0.015	0.9017	0.902
Thousand-kernel weight	*sat2* × *Rht-B1*	54.5	2.427	0.125	0.766
Test weight	*sat2* × year	528.8	1.687	0.1517	0.364
Test weight	*sat2 ×* crop management level (T1–T3, 2021–2025)	444.8	0.551	0.5768	0.956
Test weight	*sat2* × origin zone	45.4	1.553	0.2191	0.626
Test weight	*sat2* × *Rht-B1*	54.6	0.004	0.9511	0.985
Falling number	*sat2* × year	225.1	0.802	0.4497	0.600
Falling number	*sat2 ×* crop management level (T1–T3, 2021–2025)	212.1	0.305	0.7376	0.956
Falling number	*sat2* × origin zone	36.8	0.053	0.82	0.902
Falling number	*sat2* × *Rht-B1*	43.8	0.676	0.4155	0.766

**Table 5:** KASP markers of the intragenic *sat2* polymorphisms used in this study. Dye-to-nucleotide correspondence: TDsat2.i1.1, FAM = T, HEX = G; TDsat2.e9.1, FAM = G (*Gly325*), HEX = A (*Ser325*).

Marker	Position in the Gene	Nucleotide Substitution	Consequence for the Protein	Allele-Specific Primers	Dye Per Variant
TDsat2.i1.1	intron 1	G>T	—	GTTTCAGACGGCAAAGAATCCAT	Allele 1 (FAM)
				GTTTCAGACGGCAAAGAATCCAG	Allele 2 (HEX)
				GTGACTTGAAAGAAGAGAATCCAGC	
TDsat2.e9.1	exon 9	G>A	Gly325Ser	AGATTATTTCGAGCATGTTGCCG	Allele 1 (FAM)
				AGATTATTTCGAGCATGTTGCCA	Allele 2 (HEX)
				AAACTCACCGTTGGATCTACC	

**Table 6:** Two-marker haplotypes of *sat2*, their nucleotide composition and occurrence in the collection.

Haplotype	TDsat2.e9.1 (Exon 9)	Residue 325 of SAT2	TDsat2.i1.1 (Intron 1)	Cultivars in the Collection	Note
Hap1	G	Gly	T	32	higher protein content
Hap2	A	Ser	G	33	haplotype of the reference genome
Hap3	G	Gly	G	2	two cultivars (Zodiak, Lyutescens 982); too few for estimation, excluded from the models
Hap4	A	Ser	T	0	not observed

Note: Hap3 excluded from the models.

## Data Availability

The analytical data set used in all mixed models (cultivar, environment, *sat2* haplotype, and values of the twelve traits; 5913 observations, 65 cultivars) is available from the corresponding author on reasonable request. The full model outputs are provided in Supplementary Tables S1–S14.

## Supplementary Materials

The following supporting information can be downloaded at https://www.mdpi.com/article/10.3390/plants15213340/s1, Table S1, linkage between the two markers and the Hap3 accessions; Table S2, full results of the statistical models and variance components; Table S3, simple effects of the haplotype within crop management levels and the difference between extreme levels; Table S4, differences between crop management levels in grain yield and protein content within harvest years; Table S5, characterisation of the collection studied, with the call at each *sat2* marker and the haplotype of every cultivar; Table S6, stratification by origin and breeding centre; Table S7, the effect with the *Glu-A1, Glu-D1* and *1BL.1RS* covariates; Table S8, *Rht-B1* as a covariate; Table S9, structural context of the *Gly325Ser* substitution; Table S10, harvested plot areas; Table S11, growing conditions and trait coverage by harvest year; Table S12, variance components of the main models; Table S13, tests of interactions; Table S14, the effect within harvest years; Figure S1, growing conditions by agronomic year.

## Abbreviations

The following abbreviations are used in this manuscript:

SAT2	Serine acetyltransferase 2 protein
KASP-PCR	Kompetitive Allele-Specific PCR
HMW-GS	High-molecular-weight glutenin subunits
LMW-GS	Low-molecular-weight glutenin subunits
NAC	NAM/ATAF/CUC transcription factor family
Rht	Reduced height (dwarfing genes)
Gpc-B1	Grain protein content B1 (NAC transcription factor)
1BL.1RS	Translocation between chromosome 1B long arm and 1R short arm
CTAB	Cetyltrimethylammonium bromide
pLDDT	predicted Local Distance Difference Test
GOST	Russian state standards (for test methods)
VNIISB	All-Russian Research Institute of Agricultural Biotechnology
RSAU-MTAA	Russian State Agrarian University—Moscow Timiryazev Agricultural Academy
CI	Confidence interval
SE	Standard error
MDE	Minimum detectable effect (at 80% power and α = 0.05)
FDR	False discovery rate (Benjamini–Hochberg q value)
PCR	Polymerase chain reaction
Hap1–Hap4	Two-marker haplotypes of sat2 defined by the nucleotides at TDsat2.e9.1 and TDsat2.i1.1 (Table 6)
NIR	Near-infrared (spectroscopy)
OASTL	O-acetylserine (thiol) lyase
