# Supplementary material for: Effects of Serine Acetyltransferase 2 (sat2) Haplotypes and Multiple Environmental Factors on Grain Protein Content and Yield-Related Traits in Bread Winter Wheat
Source: Plants (Basel). 2026 Oct 28;15(21):3340. doi: 10.3390/plants15213340 (PMC13766025; doi:10.3390/plants15213340)
Supplement: Supplementary file 1 [file plants-15-03340-s001.zip › Figure S1.pdf]

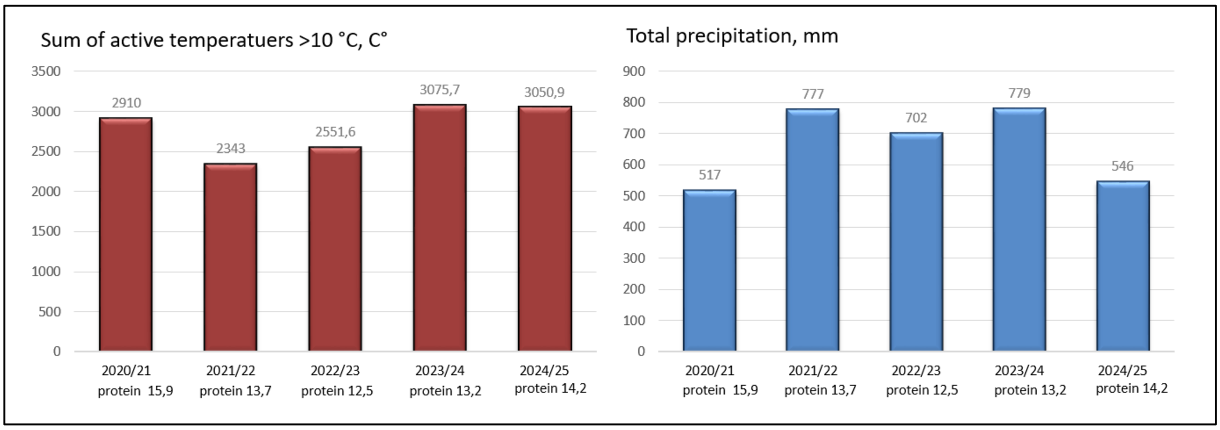

Figure S1. Growing conditions by agronomic year: (left) sum of active temperatures above 10 °C and (right) total precipitation. Below the axis, the agronomic year and the trial mean protein content for the corresponding harvest year.
